# Molecular Detection of Tick-Borne Bacterial and Protozoan Pathogens in *Haemaphysalis longicornis* (Acari: Ixodidae) Ticks from Free-Ranging Domestic Sheep in Hebei Province, China

**DOI:** 10.3390/pathogens12060763

**Published:** 2023-05-26

**Authors:** Zhongqiu Teng, Yan Shi, Na Zhao, Xue Zhang, Xiaojing Jin, Jia He, Baohong Xu, Tian Qin

**Affiliations:** 1 National Key Laboratory of Intelligent Tracking and Forecasting for Infectious Diseases, National Institute for Communicable Disease Control and Prevention, Chinese Center for Disease Control and Prevention, Beijing 102206, China; tengzhongqiu@icdc.cn (Z.T.); zhaona@icdc.cn (N.Z.); zx1127817@163.com (X.Z.); jinxiaojing9@163.com (X.J.); hejia@icdc.cn (J.H.); 2 Shijiazhuang Center for Disease Control and Prevention, Shijiazhuang 050021, China; hbsy80@126.com

**Keywords:** *Rickettsia*, *Anaplasma*, *Ehrlichia*, *Hepatozoon*, *Theileria*

## Abstract

Ticks and tick-borne pathogens significantly threaten human and animal health worldwide. *Haemaphysalis longicornis* is one of the dominant tick species in East Asia, including China. In the present study, 646 *Ha. longicornis* ticks were collected from free-ranging domestic sheep in the southern region of Hebei Province, China. Tick-borne pathogens of zoonotic and veterinary importance (i.e., *Rickettsia*, *Anaplasma*, *Ehrlichia*, *Borrelia*, *Theileria*, and *Hepatozoon* spp.) were detected in the ticks using PCR assays and sequence analysis. The prevalence rates of these pathogens were 5.1% (33/646), 15.9% (103/646), 1.2% (8/646), 17.0% (110/646), 0.15% (1/646), and 0.15% (1/646), respectively. For *Rickettsia* spp., *R. japonica* (*n* = 13), *R. raoultii* (*n* = 6), and *Candidatus* R. jingxinensis (*n* = 14) were detected for the first time in the province, while several *Anaplasma* spp. were also detected in the ticks, including *A. bovis* (*n* = 52), *A. ovis* (*n* = 31), *A. phagocytophilum* (*n* = 10), and *A. capra* (*n* = 10). A putative novel *Ehrlichia* spp. was also found with a prevalence of 1.2% in the area. The present study provides important data for effectively controlling ticks and tick-borne diseases in the Hebei Province region of China.

## 1. Introduction

Ticks are small, blood-sucking arachnids that are found throughout the world [1]. They feed on the blood of mammals, birds, reptiles, and other hosts, and in the process, can transmit various pathogens that cause disease in humans and animals [2,3,4]. There are approximately 124 species of tick found in China. Among them, *Ha. longicornis* is distributed in the northeastern, central, southern, and western regions of China [5]. Ticks are considered to be one of the most competent vectors, as they can transmit at least 15 bacterial, parasitic, and viral pathogens of zoonotic and veterinary importance [6,7]. They have become a significant public health concern due to the number of diseases they transmit, including Lyme disease, spotted fever, ehrlichiosis, and anaplasmosis [8].

Rickettsiales bacteria, including the spotted fever group of *Rickettsia* (SFGR), *Anaplasma*, and *Ehrlichia,* are recognized as important tick-borne pathogens [9,10]. Similar to its Asian neighbor countries, the major vectors of SFGR are *Dermacentor silvarum* and *Ha. longicornis* in China [11,12]. Twenty-one species of SFGR have been identified as pathogenic to humans worldwide [11]. In mainland China, at least 18 species of Candidatus species of SFGR have been referred to as human pathogens, and 8 of them have been confirmed: including *R. heilongjiangensis*, *R. japonica*, *R. raoultii*, *R. sibirica*, *R. monacensis*, *Candidatus* R. tarasevichiae, *R. XY99*, and *Ca*. R. xinyangensis [3,13,14,15,16,17,18,19,20]. In the genus *Anaplasma* and the genus *Ehrlichia,* which belong to Anaplasmatacae, *A. phagocytophilum* and *E. chaffeensis* were the causative agents of well-known tick-borne diseases: human granulocytic anaplasmosis (HGA), and human monocytic ehrlichiosis (HME), respectively. In addition, *A. bovis*, *A. capra*, *A. ovis*, *E. ewingii*, and *E. muris* have been reported to cause human infection [21,22,23,24]. *Haemaphysalis* spp. are also an important part of the natural transmission cycle of *B. burgdorferi*, a causative agent of Lyme disease [25].

Ticks are the main vector for numerous protozoan pathogens belonging to the phylum Apicomplexa, including *Babesia*, *Theileria*, and *Hepatozoon* [26]. They can infect a variety of animal hosts, including mammals and birds [27]. The parasites are transmitted through the bite of infected arthropods (especially ticks), causing significant illness in hosts. *Ha. longicornis* ticks have been implicated in the transmission of *Theileria*, *Hepatozoon*, and *Babesia* [28,29]. Though *Theileria* and *Hepatozoon* agents have not been associated with human infection in China, they inflict damage to animal husbandry production and wildlife. *Babesia* agents are significant emerging threats to animal and human health. *Ha. longicornis* ticks can act as vectors of several *Babesia* species, including human babesiosis agents, such as *Ba. microti* and *Ba. divergens* [30].

Hebei Province is located in northern China and is adjacent to Beijing and Tianjin cities. The province presents varied landscapes, including rolling hills, forests, and plains. There are several tick-borne pathogen surveillance projects in the province, mainly concentrated in the northern regions. Based on the surveillance project for tick and tick-borne pathogens of the National Institute for Communicable Disease Control and Prevention (ICDC), we found that *Ha. longicornis* is the dominant tick species in the province, especially the ticks that are parasitic on sheep. The present study aimed to investigate the prevalence and genetic diversity of the bacterial and protozoan pathogens in *Ha. longicornis* ticks on free-range sheep in Shijiazhuang City, Hebei Province, China.

## 2. Materials and Methods

### 2.1. Study Area and Tick Sampling Protocol

**Figure 1:**
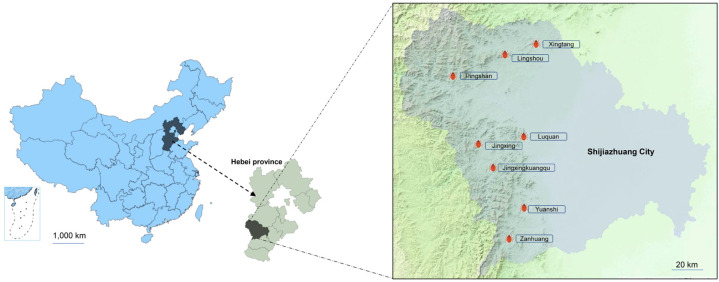
Map of the study area. Shijiazhuang City of Hebei Province, China.

This study was conducted in Shijiazhuang City in the south of Hebei Province to the east of Taihang Mountains and presents the stepped landform features, including mountains, hills, plains, and wetlands. We sampled ticks from the ears, periocular, axillary, and neck of free-ranging sheep (Figure 1) in Pingshan, Luquan, Jingxing, Jingxing Mining District, Yuanshi, Lingshou, Xingtang, and Zanhuang counties in the spring and autumn of 2022. Tick species were first identified morphologically using taxonomic keys and then confirmed by nested PCR amplification and sequencing of the *CO1* genes (Table S1) [31,32].

### 2.2. DNA Extraction 

All the ticks were washed with bromogeramine (5%), alcohol, and water, respectively, for 15 min each. After air-drying, the ticks were individually homogenized, and then the DNA was extracted following the protocol of the QIAamp DNA Mini Kit (Qiagen, Germany). 

### 2.3. Detection of Bacteria and Parasites in Ticks

Bacterial pathogens, including *Rickettsia* spp., *Anaplasma* spp., *Ehrlichia* spp., *Bartonella* spp., *Borrelia* spp., *C. burnetii*, and *F. tularensis,* were screened using real-time PCR (qPCR) with the corresponding primers described in Table S1. The tick DNA samples positively detected in the rickettsia-specific qPCR test (CT value < 38) were further tested using nested PCR targeting an 1100 bp region of the *glt*A gene, a 440 bp region of the 17 kD gene, a 530 bp sequence of the *omp*A gene, and a 1200 bp region of the *rrs* gene of SFGR. The tick DNA samples testing positive for Anaplasmataceae in qPCR (CT value of <38) were confirmed and preliminarily typed using nested PCR targeting a 500 bp region of the *rrs* gene that could amplify both *Anaplasma* spp. and *Ehrlichia* spp. A set of genus-specific or species-specific primers of *Anaplasma* and *Ehrlichia* targeting the *rrs*, *glt*A, and *groEL* genes (heat shock protein) were used for the identification of bacterial species and phylogenetic analysis. For the putative novel *Ehrlichia* strains, sequences of *ftsZ* (cell division protein gene), conP28 (P28 major membrane protein gene), and *dsb* (disulfide oxidoreductase) genes were also obtained with amplification and sequencing. *Borrelia-*positive samples were reevaluated by nested PCR targeting the 350 bp *osp*A (outer surface protein A) gene.

For the detection and characterization of tick-borne protozoan pathogens, nested PCR was performed using a universal primer set targeting the 18S rRNA gene of *Babesia–Theileria–Hepatozoon* [33].

The target amplicons were isolated with the QIAquick PCR Purification Kit (Qiagen, Hilden, Germany) and then sent to Beijing De’aoping Biotechnology Co., Ltd. (Beijing, China), for sequencing. The PCR primers are shown in Table S1. The agarose gel electrophoresis images of representative isolates in each pathogen are provided in Supplementary Data.

### 2.4. Phylogenetic Data Analysis

For the *rrs* genes of *Rickettsia*, *Anaplasma* spp., and *Ehrlichia* spp., two overlapping fragments were first edited and assembled using SeqMan software (DNASTAR, Madison, WI, USA) to obtain the almost complete gene sequences. Qualified and trimmed sequences were identified by comparison with the sequences available in GenBank with the Basic Local Alignment Search Tool (BLAST) (https://blast.ncbi.nlm.nih.gov/Blast.cgi accessed on 28 March 2023). Phylogenetic and molecular evolutionary analysis was performed using the neighbor-joining method with 1000 replicates for bootstrap analysis in MEGA 7.0 (https://www.megasoftware.net accessed on 28 March 2023).

### 2.5. Accession Numbers of Nucleotide Sequence 

The sequences obtained in this study were deposited into GenBank with the accession numbers: ticks (OQ699158-OQ699195), bacteria (OQ701062-OQ701079, OQ702255-OQ702302), and protozoan (OQ695453-OQ695455).

## 3. Results

### 3.1. Tick Sampling and Identification

A total of 646 adults ticks (29 fully engorged ticks, 131 partially engorged, and 486 unfed ticks) were collected from free-ranging sheep in the Shijiazhuang City in the south of Hebei Province (Figure 1). All collected ticks were identified as *Ha. longicornis* based on morphology. *CO1* gene sequences of the collected ticks were acquired by amplifying and sequencing (Table S1) those sharing 99–100% identity with the sequences of *Ha. longicornis* from GenBank (MK450606).

### 3.2. Tick-Borne Pathogens Detected

For the detection of bacterial pathogens using the PCR assays, *Rickettsia* spp., *Anaplasma* spp., *Ehrlichia* spp., and *Borrelia* spp. were detected in the ticks, while *Bartonella* spp., *F. tularensis*, and *C. burnetii* were not detected (Table 1). Among a total of 646 *Ha. longicornis* ticks, 33 (5.1%) ticks were positive for *Rickettsia* spp., including 13 (2.0%) ticks that were infected with *R. japonica*, 6 (0.9%) ticks with *R. raoultii*, and 14 (2.2%) ticks with *Ca.* R. jingxinensis, while 103 (15.9%) ticks were infected with *Anaplasma* spp., including 52 (8.0%) ticks infected with *A. bovis*, 31 (4.8%) ticks with *A. ovis*, 10 (1.5%) ticks with *A. capra*, and 10 (1.5%) ticks with *A. phagocytophilum*. In addition, eight (1.2%) ticks were infected with *Ehrlichia* spp. *Borrelia burgdorferi* was detected in only one tick.

Based on the amplification and sequencing of piroplasms’ 18S rRNA gene, 110 (17.9%) ticks were infected with *T. luwenshuni*, and only 1 (0.15%) tick was infected with *H. felis*, but *Babesia* spp. were not detected among the collected ticks.

Co-infection with two or three tick-borne pathogens within an individual tick was detected in 42 (6.5%) of the ticks tested. Two (0.3%) ticks were co-infected with *Anaplasma* spp. and *Rickettsia* spp., seven (1.1%) ticks were co-infected with *Rickettsia* spp. and *Theileria* spp.), twenty-seven (4.2%) ticks were co-infected with *Anaplasma* spp. and *Theileria* spp., and only one tick was co-infectedwith *Ehrlichia* spp. and *Hepatozoon* spp.

### 3.3. Phylogenic Analysis of Different Tick-Borne Pathogens

**Figure 2:**
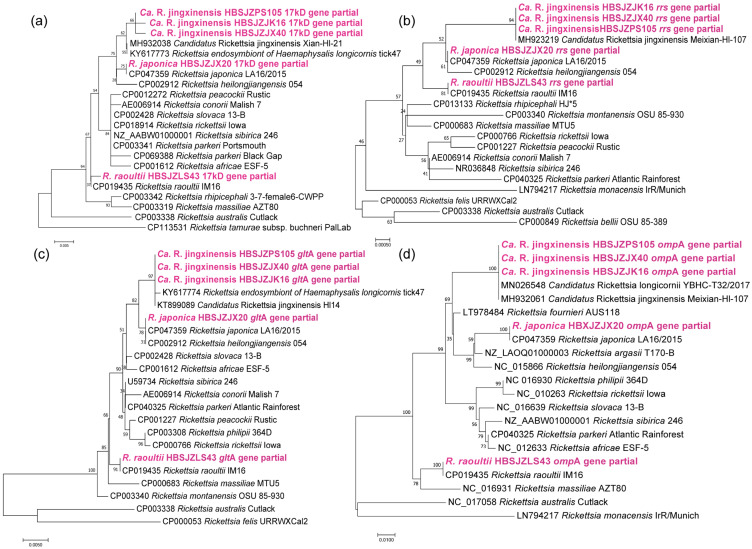
Phylogenetic analysis of *Rickettsia* strains based on the nucleotide sequences of 17 kD (440 bp), *rrs* (1200 bp), *glt*A (900 bp), and *omp*A (500 bp) found in ticks using the maximum likelihood method with 1000 bootstraps: (**a**). 17 kD gene; (**b**). *rrs* gene; (**c**). *glt*A gene; (**d**). *omp*A gene.

*Rickettsia*: Phylogenetic analysis based on the *rrs*, *glt*A, *omp*A, and 17 kD genes showed that three *Rickettsia* spp. identified in the ticks clustered together with *R. japonica*, *R. raoultii*, and *Ca.* R. jingxinensis (Figure 2). The sequences of *rrs*, *glt*A, *omp*A, and 17 kD genes for *R. japonica* and *rrs*, *glt*A, and *omp*A for *R. raoultii* were identical to *R. japonica* (CP047359) and *R. raoultii* (CP019435). The 17kD gene of the obtained *R. raoultii* strain was 99.42% similar to *R. raoultii* (CP019435). For the samples HBSJZJX40, HBSJZJK16, and HBSJZPS105, which clustered closely with *Ca.* R. jingxinensis, the *rrs*, *omp*A, and *glt*A gene sequences obtained from the ticks showed 100% identity, and the 17 kD gene sequence showed 99.28–99.76% identity to *Ca.* R. jingxinensis (MH932038) or *Ca*. R. longicornii (KY617773). 

**Figure 3:**
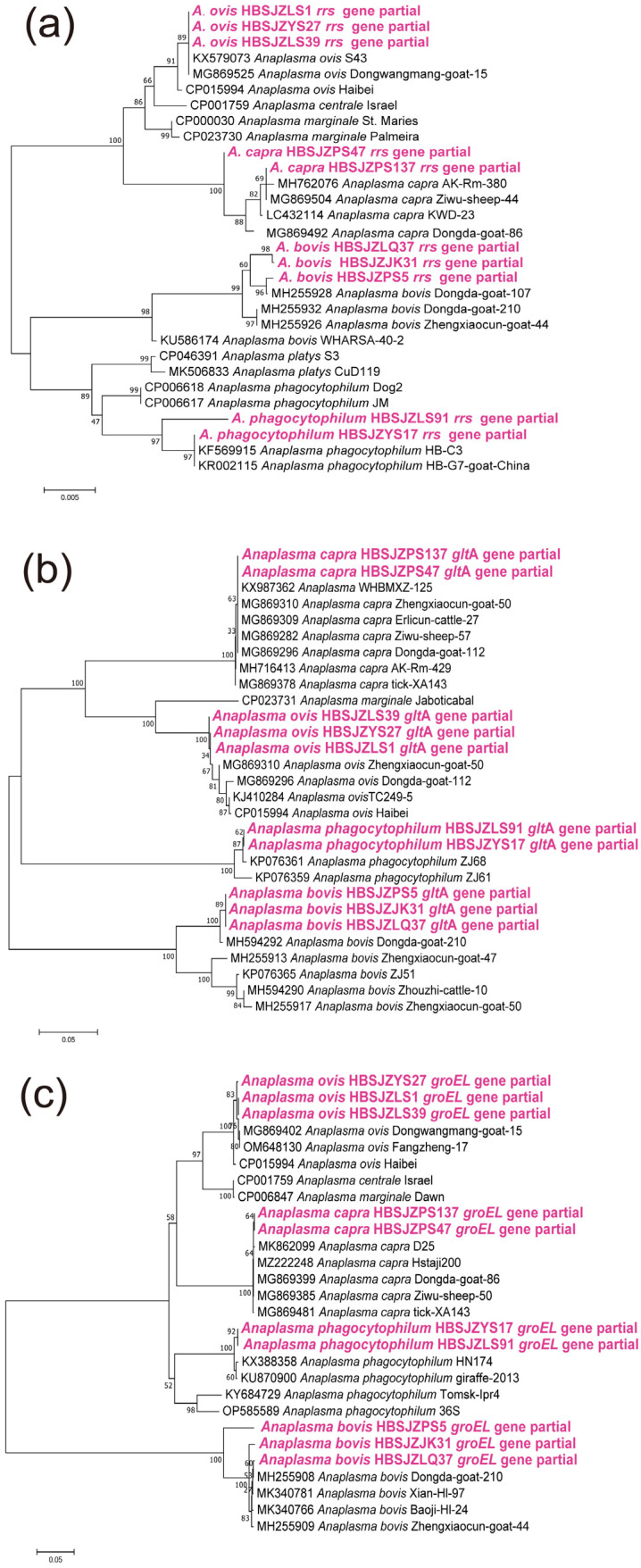
Phylogenetic analysis of *Anaplasma* strains based on the nucleotide sequences of *rrs* (1400 bp), *glt*A (400 bp), and *groEL* (330 bp) genes found in ticks using the maximum likelihood method with 1000 bootstraps: (**a**). *rrs* gene; (**b**). *glt*A gene; (**c**). *groEL* gene.

*Anaplasma*: Four *Anaplasma* species (*A. bovis*, *A. ovis*, *A. phagocytophilum*, and *A. capra*) were identified in the ticks. *A. bovis* detected in the current study were classified into three genotypes in the phylogenetic tree based on the *rrs*, *glt*A, and *groEL* genes. The sequences of the three genes shared 99.77–100% identity with *A. bovis* strains from other provinces of China. All *rrs* gene sequences of *A. ovis* obtained in this study were identical to each other, and the isolates were closely related to *A. ovis* isolates from goats (MG869525) and sheep (KX579073) in China. Sequences of the *glt*A and *groEL* genes for the three *A. ovis* isolates showed 99.97% and 99.51 to 100% intersequence identities, since they are still closely related to *A. ovis* strains in Shannxi Province (*glt*A: MG869310 and MG869296; *groEL:* MG869402 and OM648130). The partial *rrs* gene sequences of *A. phagocytophilum* identified in sheep were 99.83 to 99.91% identical to the isolates derived from *Ha. longicornis* (KF569915) and goat (KR002115). The *glt*A and *groEL* sequences were closely related to *A. phagocytophilum* strains reported in China (KP076361 and KX388358), with similarities of 99.12% and 99.28%. For the two isolates of *A. capra*, the *rrs*, *glt*A, and *groEL* sequences had 99.91 to 99.93%, 100%, and 99.86% identity with those of reported *A. capra* strains (MH762076, KX987362, and MG869399), respectively (Figure 3). 

**Figure 4:**
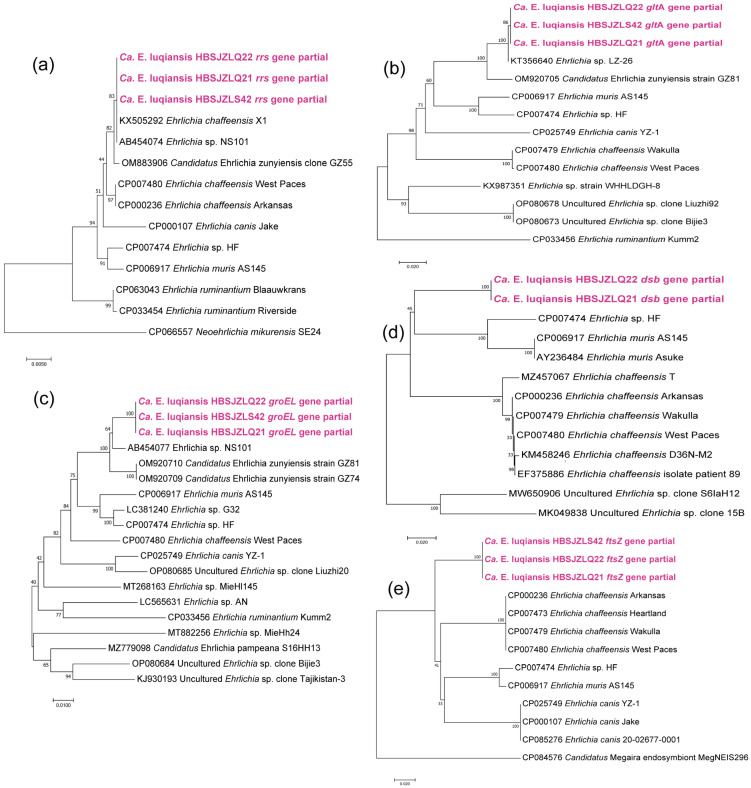
Phylogenetic analysis of *Ehrlichia* strains based on the nucleotide sequences of *rrs* (1250 bp), *groEL* (1109 bp), *glt*A (800 bp), *dsb* (300 bp), and *ftsZ* (400 bp) genes using the maximum likelihood method with 1000 bootstraps: (**a**). *rrs* gene; (**b**). *glt*A gene; (**c**). *groEL* gene; (**d**). *dsb* gene; (**e**). *ftsZ* gene.

*Ehrlichia*: PCR detection indicated that eight ticks were infected with *Ehrlichia* spp. The sequences of nearly complete *rrs* genes showed 100% identity to *E.* sp. NS101 (AB454074) or *E. chaffeensis* isolate X1 (KX505292) and 99.60% identity to other strains of *E. chaffeensis* (query cover: 100%; E-value: 0.0). However, the partial *glt*A and *groEL* sequences of the *Ehrlichia* spp. share 86.17 to 86.28% and 94.33% identity with the *E. chaffeensis* strains (Query cover: 100%, E-value: 0.0). The obtained *groEL* sequences were 98.26% similar to *E.* sp. NS101, but the *glt*A gene records were absent for the NS101 strain. Although these strains were closest to the *Candidatus* Ehrlichia zunyiensis found in Guizhou Province in the phylogenetic trees of the *glt*A and *groEL* genes, the identity similarity was only 97.33% and 96.47% for the two single genes. The sequences of partial *ftsZ*, conP28, and *dsb* genes of the *Ehrlichia* spp. were also obtained and deposited in Genbank. The *dsb* and *ftsZ* genes of the *Ehrlichia* strains detected in the present study had 85 to 85.33% (query cover: 100%; E-value: 0.0) and 88.31 to 88.57% (query cover: 99%; E-value: 0.0) identity to *E. chaffeensis* (Figure 4). We therefore propose that they represent a novel species, and we name it “Candidatus Ehrlichia luquansis” according to the site where they were detected. 

**Figure 5:**
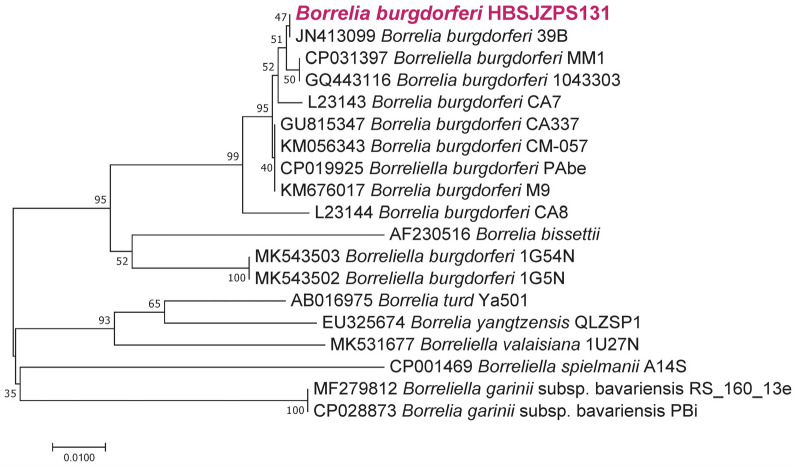
Phylogenetic analysis of the *Borrelia* strain based on the nucleotide sequences of the *osp*A (500 bp) gene found in ticks using the maximum likelihood method with 1000 bootstraps.

*Borrelia*: Based on the *osp*A gene, one tick sample shared complete nucleotide identity with *Borrelia.* Phylogenetic analysis based on the *osp*A gene showed that the *Borrelia* strain clustered together with *B. burgdorferi* (JN413009) (Figure 5). 

**Figure 6:**
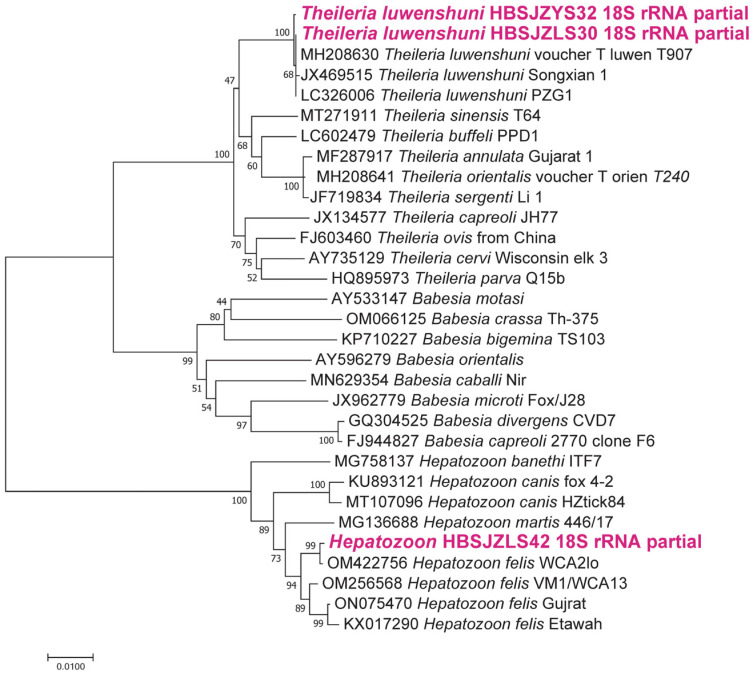
Phylogenetic analysis of the *Theileria and Hepatozoon* strains based on the nucleotide sequences of the 18S rRNA (1431 bp) gene found in ticks using the maximum likelihood method with 1000 bootstraps.

Protozoan: Two sequences of the 18S rRNA gene exhibiting 99.98% intersequence identities were identified as belonging to *Theileria* strains in the ticks. As shown in Figure 6, the 18S rRNA gene sequences of the *Theileria* strains detected in this study showed 99.3% to 100% identity to *T. luwenshuni* (MH208630) that was detected in the *Rh. microplus* ticks in China and had 99.79 to 99.86% identity to *T. luwenshuni* (JX469515) from small ruminants in China. Sequencing and BLAST analysis revealed that a sequence of the 18S rRNA gene was highly similar to the gene of *H. felis* isolated from wildcats (*Felis silvestris*) in Hungary (OM256568 and OM256569) and from an Asiatic lion in India (ON075470 and KX017290) with 98.68% and 99.85% identities, respectively. 

## 4. Discussion

*Ha. longicornis*, also named the Asian longhorned tick, is a human-biting Ixodidae tick species native to East Asia, especially in eastern China, Japan, and Korea. However, the distribution regions of the ticks have expanded to Australia, New Zealand, and the USA [7,34]. In China, *Ha. longicornis* is the most prevalent tick species, distributed through at least 17 provinces [31], and it is regarded as an important vector of infectious diseases threatening human and animal health, due to its broad host range, diverse vegetation habitats, and multiple pathogens associated with a wide spectrum of human and animal diseases [35]. In this study, SFG *Rickettsia* (*R. japonica*, *R. raoultii*, and *Ca.* R. jingxinensis), *Anaplasma* spp. (*A. phagocytophilum*, *A. bovis*, *A. ovis,* and *A. capra*), *Ehrlichia* spp., *B. burgdorferi*, *T. luwenshunni*, and *H. felis* were identified in *Ha. longicornis* ticks which were collected from free-ranging sheep in eight counties along the eastern side of the Taihang Mountains in southern Hebei Province. 

*Ha. Longicornis* were reported as vectors of several *Rickettsia* spp. (*R. raoultii*, *R. japonica*, *R. heilongjiangensis*, *Ca.* R. tarasevichiae, *Ca.* R. jingxinensis, *Ca.* R. jiaonani, and *Ca.* R. hebeiii) in China [6]. In the present work, *R. japonica*, *R. raoultii* and *Ca.* R. jingxinensis were first detected in the ticks of Hebei Province in the present surveillance. *R. japonica* was the causative agent of Japanese spotted fever (JSF). The pathogen was first described in Japan and human cases were found in Japan, South Korea, the Philippines, Thailand, and China [15,36,37]. In recent years, human JSF cases have been found in Zhejiang, Anhui, Hubei, and Henan provinces of China [38,39,40], and two fatal cases occurred in Hubei province [15]. *R. japonica* has been detected in ticks from many provinces in northern, eastern, central, and northeastern China [6,41]. *R. raoultii*, a causative agent of tick-borne lymphadenopathy in humans was first described in the *D. nuttalli* ticks in Siberia and the *Rhipicephalus pumilio* ticks in the Astrakhan region in 1999 [42]. Human infections with *R. raoultii* were first confirmed in Spain and have since been reported in several provinces of China [43,44]. Although Dermacentor ticks were considered to be the dominant vectors of *R. raoultii*, it was also detected in other ticks, including *Ha. erinaceid*, *Ha. concinna*, *Ha. qinghaiensis*, and *Ha. longicornis* [45,46]. *Ca.* R. jingxinensis is a novel *Rickettsia* species with potential pathogenicity that has been reported to be widespread in China and co-circulates in various ticks [47]. To date, no human cases of SFGR infection have been reported in Hebei Province, but the presence of *R. raoultii* and *R. japonica* suggests a risk of Rickettsial infection in local residents. A putative novel *Rickettsia* spp., named *Ca.* R. hebeiii was previously reported in ticks with a minimum prevalence of 0.7% in the ticks in the province, but it was not detected in the present study [48]. Ticks and the pathogens that they carry can exhibit temporal variations, with changes in their distribution and prevalence occurring over time. 

A high diversity of *Anaplasma* spp. were found in the *Ha. longicornis* ticks in this study. The presence of *A. phagocytophilum*, *A. bovis*, *A. ovis*, and *A. capra* was detected in the ticks, and the infection rate of *A. bovis* (8.0%) and *A. ovis* (4.8%) were the highest, suggesting that *A. bovis* and *A. ovis* were the dominant species of the genus *Anaplasma,* which is prevalent in *Ha. longicornis* ticks in Hebei Province. Interestingly, most *A. ovis* strains were detected in ticks collected in Pingshan county, indicating the potential differences in the geographical distribution of *A. ovis*, as it was not detected in the ticks from the adjacent counties, even though the adjacent regions have similar ecological environments. *A. bovis* was initially found as a pathogen of cattle but has been reported to be present across a broad host range [21]. Three genotypes of *A. bovis* were found in the present study, demonstrating its diversity in the ticks of Hebei province. *A. ovis* is widely distributed in North America, Asia, Africa, and Europe [49]. Sheep and goats are the main hosts, and livestock infection can lead to the loss of the local pasturage economy. *A. capra*, a zoonotic pathogen, was also detected in the present study, with a minimum infection rate of 1.5%. In addition, *A. bovis* and *A. ovis* can also cause human infection. Though the prevalence of *A. phagocytophilum* was 1.5% in the ticks, lower than that of others, it is a well-known tick-borne pathogen causing HGA. HGA cases and a high prevalence of antibodies to *A. phagocytophilum* in local residents were found in different regions of China [50,51,52]. Attention should be paid to the risks of HGA for local residents in Hebei Province.

A putative novel species of the genus *Ehrlichia* was detected in the ticks in the present study. The highest degree of identities of *rrs*, *glt*A, *groEL*, *dsb*, and *ftsZ* amplified from the novel species in the ticks were 100%, 96.47%, 98.26%, 85.33%, and 88.57%, respectively, compared with those from known *Ehrlichia* species. The species was close to *Ca.* E. zunyiensis which was detected in *Berylmys bowersi* in Guizhou Province, China, and *Ehrlichia* sp. NS101, which was identified in deer in Japan. Similarly, the *rrs* genes of these species were most closely related to that of *E. chaffeensis*, but other test genes were not. There may be a cluster of *Ehrlichia* spp. with similar *rrs* genes, but which are diverse in genomes widely distributed in East Asia. This result suggests that multiple genes should be analyzed in the genotyping of the *Ehrlichia* species. The potential pathogenicity of the *Ehrlichia* species needs to be further studied.

*B. burgdorferi* was also detected in the *Ha. longicornis* ticks with a low prevalence (0.2%) in the present study. *Ixodes persulcatus* and *Ha. japonica* ticks are recognized as the primary vector of *B. burgdorferi* in northern China [25]. In a previous study, 17.14% of *I. persulcatus* and 10% of *Ha. japonica* ticks were positively detected in PCR targeting the *B. burgdorferi* gene, but all of the *Ha. longicornis* ticks were negative [53]. However, *B. burgdorferi* strains were isolated from *Ha. longicornis* ticks in Beijing, which is surrounded by Hebei [54]. Our results indicated that the *Ha. longicornis* ticks in the investigated sites can serve as a vector of *B. burgdorferi.*

*T. luwenshuni* and *H. felis* were detected in the *Ha. longicornis* ticks using PCR targeting *Babesia*, *Theileria*, and *Hepatozoon* 18sRNA, with a 17.9% and 0.15% prevalence, respectively. *T. luwenshuni* can be transmitted by *Ha. qinghaiensis* and *Ha. longicornis* ticks, which are mainly reported in northwestern regions of China. Our results agree and suggest that *Ha. longicornis* acts as a vector *of T. luwenshuni.* The pathogen can cause theileriosis that affects domestic and wild ruminants, including sheep, goats, cattle, and deer. *T. luwenshuni* is transmitted to animals through the bite of infected ticks, causing a range of symptoms, including fever, anemia, and weight loss in livestock, especially goats and sheep, and even causing death in serious cases. An *Ha. longicornis* tick was shown to be positive for *H. felis*, a pathogen to felids. It can infect hosts via the bite of ticks or infected prey. Our study provides evidence that *Ha. longicornis* may be a biological vector of *H. felis* in Hebei Province and poses threats to wild felids and domestic cats with field contact.

The present study does have limitations. The PCR-positive detection of pathogens in the collected parasitic ticks from sheep cannot differentiate whether the pathogens’ DNA templates were from infected ticks or sheep blood that was degraded in tick guts. The investigation of tick-borne pathogens in local sheep and free ticks should be carried out in further studies.

## 5. Conclusions

In summary, we identified numerous bacterial and protozoan pathogens in *Ha. longicornis* ticks from free-ranging domestic sheep in Hebei Province. *R. japonica* and *R. raoultti*, the agents of spotted fever, were first detected in the province. A high diversity of pathogens belonging to Anapasmatacae, including a putative novel *Candidatus Ehrlichia* spp., were found harboring in *Ha. longicornis* ticks. In addition, it was determined that protozoan pathogens that can infect wild and domestic animals were found with a high prevalence. The results indicate that tick-borne diseases are a threat to public health and animal husbandry in the region. Due to the constantly changing climate, environment, and human activities affecting the prevalence of ticks and their vector pathogens, surveillance of tick-borne pathogens is required for developing new control strategies.

## Figures and Tables

**Table 1:** Prevalence of tick-borne pathogens in 646 *Haemaphysalis longicornis* ticks collected from sheep in Hebei, China.

Pathogen Species		Prevalence (%) *
*Rickettsia*		5.1%
	*R. japonica*	2.0%
	*R. raoultii*	0.9%
	*Ca.* R. jingxinensis	2.2%
*Anaplasma*		15.9%
	*A. bovis*	8.0%
	*A. ovis*,	4.8%
	*A. phagocytophilum*	1.5%
	*A. capra*	1.5%
*Ehrlichia*		1.2%
	*Ca*. E. luquansis	1.2%
*Borrelia*		0.15%
	*B. burgdorferi*	0.15%
*Theileria*		17.9%
	*T. luwenshuni*	17.9%
*Hepatozoon*		0.15%
	*H. felis*	0.15%

* Total infection comprises cases of coinfection with tick-borne pathogens.

## Data Availability

All relevant data are within the paper and its supporting information files.

## Supplementary Materials

The following supporting information can be downloaded at: https://www.mdpi.com/article/10.3390/pathogens12060763/s1, Table S1. Primers for the amplification of sequences of ticks and tick-borne pathogens [13,33,53,55,56,57,58,59,60,61,62,63,64,65,66,67,68,69,70,71]; Supplementary Data. The electrophoretic detection of amplification of tick-borne pathogens PCR products on agarose gel.
