# Supplementary material for: Molecular Detection of Tick-Borne Bacterial and Protozoan Pathogens in Haemaphysalis longicornis (Acari: Ixodidae) Ticks from Free-Ranging Domestic Sheep in Hebei Province, China
Source: Pathogens. 2023 May 26;12(6):763. doi: 10.3390/pathogens12060763 (PMC10303831; doi:10.3390/pathogens12060763)
Supplement: Supplementary file 1 [file pathogens-12-00763-s001.zip › Supplementary data.pdf]

Supplementary Data

The Electrophoretical detection of amplification of tick-borne pathogens PCR products on agarose gel.

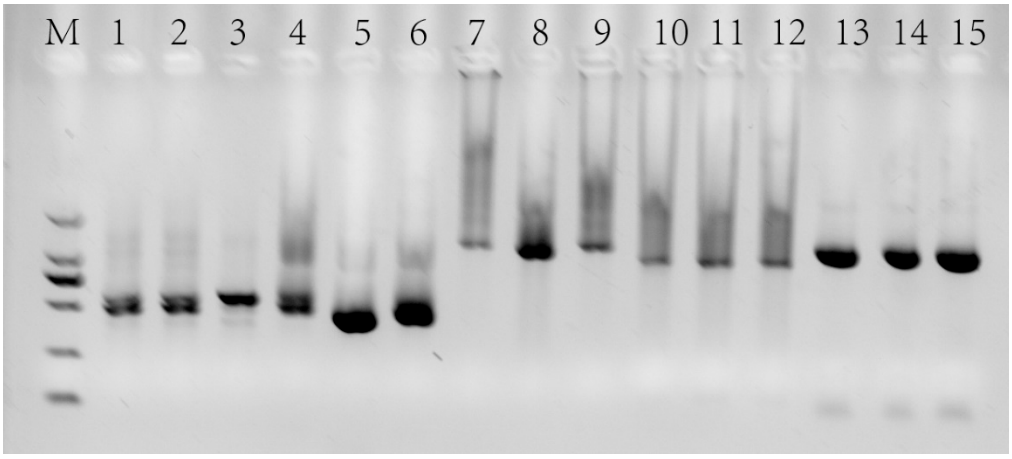

Figure S1. Electrophoretical detection of amplification of *Rickettsia* PCR products on agarose gel.
M: 100-2000 bp marker (100, 250, 500, 750, 1000, 2000 bp); line 1-line3: *omp*A genes; line 4-line 6: 17 kD genes; line 7-line 9: *glt*A genes; line 10-line 12: *rrs* gene fragments 1; line 13-line 15: *rrs* gene fragments 2

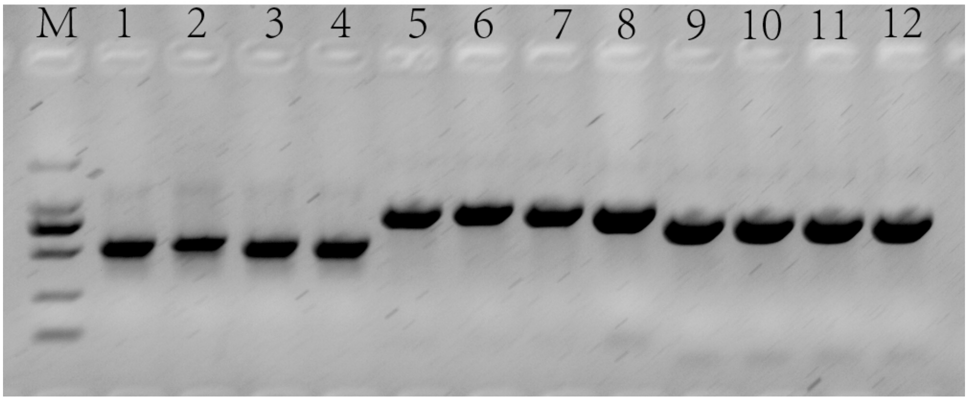

Figure S2. Electrophoretical detection of amplification of *Anaplasma rrs* genes PCR products on agarose gel.
M: 100-2000 bp marker (100, 250, 500, 750, 1000, 2000 bp); line 1-line4: 500 bp *rrs* gene fragments for screening; line 5-line 8: *rrs* gene fragments 1; line 9-line 12: *rrs* gene fragments 2.

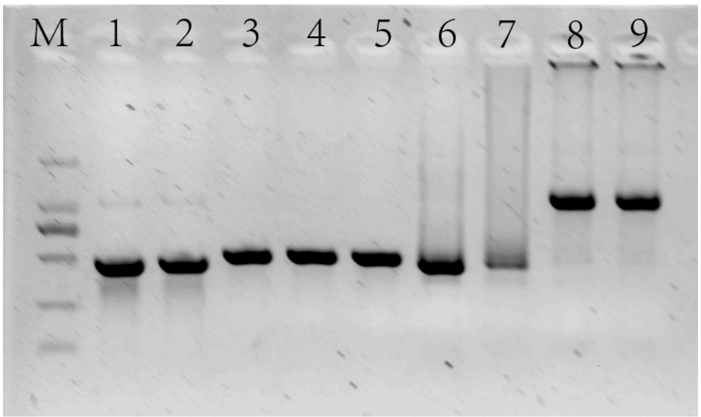

Figure S3. Electrophoretical detection of amplification of *Anaplasma glt*A genes PCR products on agarose gel.

M: 100-2000 bp marker (100, 250, 500, 750, 1000, 2000 bp); line 1-line2: *A. phagocytophilum;* line 3-line 5: *A. ovis*; line 6-line 7: *A.capra*; line 8-line 9: *A. bovis.*

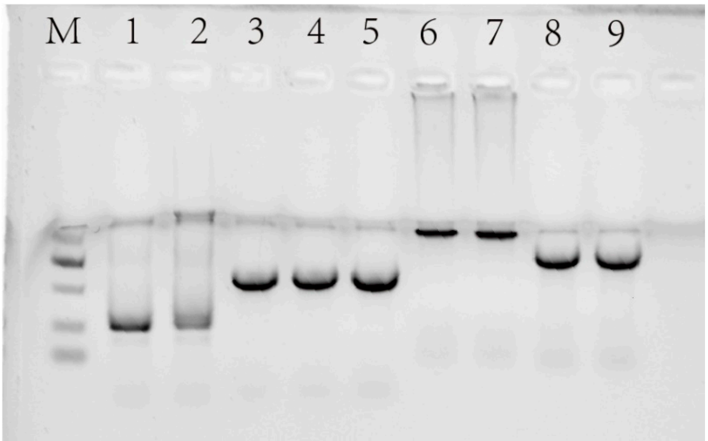

Figure S4. Electrophoretical detection of amplification of *Anaplasma groEL* genes PCR products on agarose gel.

M: 100-2000 bp marker (100, 250, 500, 750, 1000, 2000 bp); line 1-line2: *A. phagocytophilum*; line 3-line 5: *A. ovis*; line 6-line 7: *A. capra*; line 8-line 9: *A. bovis.*

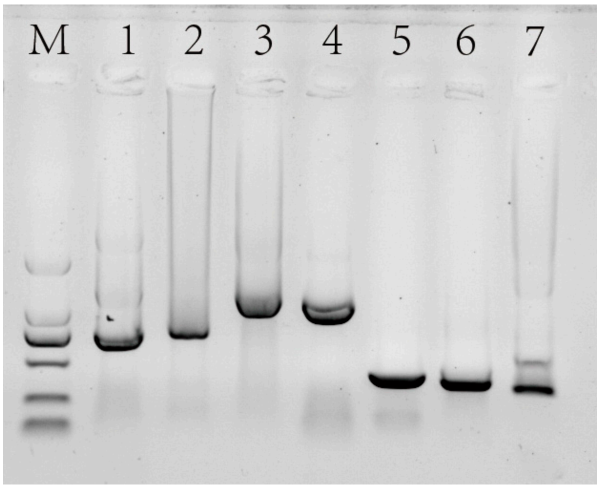

Figure S5. Electrophoretical detection of amplification of *Ehrlichia* PCR products on agarose gel.

M: 100-2000 bp marker (100, 250, 500, 750, 1000, 2000 bp); line 1: *rrs* gene fragment 1; line 2: *rrs* gene fragment 2; line 3: *glt*A gene; line 4: *groEL* gene; line 5:*dsb* gene; line 6: *ftxZ* gene; line 7: conP28 gene.

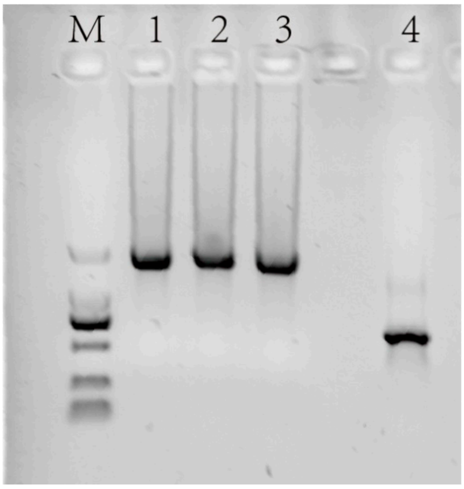

Figure S6. Electrophoretical detection of amplification of Protozoan Pathogens and *Borrelia* PCR products on agarose gel.

M: 100-2000 bp marker (100, 250, 500, 750, 1000, 2000 bp); line 1-2: *Theileria luwenshuni* 18S rRNA genes; line 3: *Hepatozoon felis* 18S rRNA genes; line 4: *Borrelia burgdorferi osp*A gene.
