# Supplementary material for: Molecular Detection of Tick-Borne Bacterial and Protozoan Pathogens in Haemaphysalis longicornis (Acari: Ixodidae) Ticks from Free-Ranging Domestic Sheep in Hebei Province, China
Source: Pathogens. 2023 May 26;12(6):763. doi: 10.3390/pathogens12060763 (PMC10303831; doi:10.3390/pathogens12060763)
Supplement: Supplementary file 1 [file pathogens-12-00763-s001.zip › TableS1 new2.pdf]

Table S1. Primers for the amplification of sequences of ticks and tick-borne pathogens

Organism	PCR method	Gene	Primer name	Sequences	References
ticks	nested PCR	*CO1*	TCOIF1*	GGAGCYCCWGATATAGCTTTCCC	[55]
			TCOIR1*	CCTGGTAAAATTAAAATATAAACTTC	
			TCOIF2#	TTTTACCGCGATGAHTWTTYT	
			TCOIR2#	WGGRTGRCCAAARAATCAAAATA	
Rickettsia	qPCR	*rrs*	R-rrs -F	GGAGCATGCGGTTTAATTCG	[56]
			R -rrs-R	GCCATGCAACACCTGTGTGT	
			R -rrs-Probe	VIC-CGGATCGCAGAGATG-MGB	
	semi-nested PCR	*rrs* fragment 1	R-rrs1-147F*#	GTACGGAATAACTTTTAGAAAT	[57]
			R-rrs1-1175R1*	CATGATGACTTGACRTCGT	
			R-rrs1-1034R2#	CATCTCACGACACGAGCTG	
		rrs fragment 2	R-rrs2-693F1*	GAAGGCGRTCATYTRGGCT	
			R-rrs2-699F2#	GRTCATYTRGGCTRCAACTG	
			R-rrs2-1427R*#	CTGCCTCTTGCGTTAGCT	
	semi-nested PCR	*glt*A	R-gltA-T151F1*	CCGGGYTTTATGTCTACTGC	[57]
			R-gltA-T156F2#	CTTTATGTCTACTGCKTCTTG	
			R-gltA-T1239R*#	AGCTGTCTWGGTCTGCTGATT	
	nested PCR	17kD	R-r17-F1*	TTTACAAAATTCTAAAAACCAT	[13]
			R-r17k-R1*	TCAATTCACAACTTGCCATT	
			R-r17k-F2#	GCTCTTGCAACTTCTATGTT	
			R-r17k-R2#	TCAATTCACAACTTGCCATT	
	Semi-nested PCR	*omp*A	R-r190k-F*#	TGGCGAATATTTCTCCAAAA	[58,59]
			R-r190k-R1*	TGCATTTGTATTACCTATTGT	
			R-r190k-R2#	AGTGCAGCATTCGCTCCCCCT	

Anaplasmataceae	qPCR	23S rRNA	Ana-F	TGA CAG CGT ACC TTT TGC AT	[60]
			Ana-R	GTA ACA GGT TCG GTC CTC CA	
			Ana-Probe	GGA TTA GAC CCG AAA CCA AG	
	nested PCR	*rrs*	A&E-rrs-F1*	GAA CGA ACG CTG GCG GCA AGC	[61]
			A&E-rrs-R1*	AGT A(T/C)C G(A/G)A CCA GAT AGC CGC	
			A&E-rrs-F2#	TGC ATA GGA ATC TAC CTA GTA G	
			A&E-rrs-R2#	CTA GGA ATT CCG CTA TCC TCT	
Anaplasma spp.	semi-nested PCR	*rrs* fragment 1	ANArrs1-F1*#	GGATAGCCACTRGAARTGGTG	[62]
			ANArrs1-R1*	CGTGCTGACTTGACATCAT	
			ANArrs1-R2#	CATCTCACGACACGAGCTG	
	semi-nested PCR	*rrs* fragment 2	ANArrs2-F1*	CTGTCTGGTCCGGTACTGAC	
			ANArrs2-F2#	TGGTCCGGTACTGACRCT	
			ANArrs2-R1*#	TGCCTCCTTDCGGTTGGC	
	nested PCR	*glt*A	ACS-F1*	GCGATTTTAGAGTGYGGAGATTG	[62]
			ACS -R1*	TACAATACCGGAGTAAAAGTCAA	
			ACS-F2#	GGGTTCMTGTCYACTGCTGCGTG	
			ACS -R2#	TTGGATCGTARTTCTTGTAGACC	
	Nested PCR	*groEL*	HS1-F*	CGYCAGTGGGCTGGTAATGAA	[63]
			HS6-R*	CCWCCWGGTACWACACCTTC	
			HS3-F#	ATAGTYATGAAGGAGAGTGAT	
			HSV-R#	TCAACAGCAGCTCTAGTWG	
A. bovis	semi-nested PCR	*groEL*	Ab gro-ELF1*	GTTCGCAGTATTTTGCCAGT	[64]
			Ab gro-ELF2#	ATCTGGAAGRCCACTATTGAT	
			Ab gro-ELR*#	CTGCRTTCAGAGTCATAAATAC	
A. capra	nested PCR	*groEL*	Ac gro-ELF1*	GCGAGGCGTTAGACAAGTCCATT	[65]
			Ac gro-ELR3*	TCCAGAGATGCGAGCGTGTATAG	

			Ac gro-ELF2#	TGCACTGCTGGTCCAAAGGGGCT	
			Ac gro-ELR2#	CAACTTCGCTAGAGCCGCCAACC	
A. phagocytophilum	semi-nested PCR	*groEL*	Ap&p groELF*#	ATGGTATGCAGTTTGATCGC	[65]
			Ap&p gro-ELR1*	TCTACTCTGTCTTTGCGTTC	
			Ap gro-ELR2#	TTGAGTACAGCAACACCACCGGAA	
Ehrlichia spp.	semi-nested PCR	*rrs* fragment 1	EHRrrs1-F1*#	GAATAGCCATTAGAAATGATG	[62]
			EHRrrs1-R1*	GTCAGTATCGAACCAGATAG	
			EHRrrs1-R2#	GTATCGAACCAGATAGCCG	
		rrs fragment 2	EHRrrs2-F1*	CGGCTATCTGGTTCGATAC	
			EHRrrs2-F2#	CTATCTGGTTCGATACTGAC	
			EHRrrs2-R1*#	GCTTCCTTKCGGTTAGCAC	
	nested PCR	*glt*A	ECS-F1*	TATGRTCRAAGAAGCAGTATT	[66]
			ECS-R1*	GGAATATTAACTTATGATCC	
			ECS-F2#	CTGACGTGGACGACATATCT	
			ECS-R2#	TGGGCTGGYAATGAAATTGA	
	nested PCR	*groEL*	HS1-F*	CGYCAGTGGGCTGGTAATGAA	[67]
			HS6-R*	CCWCCWGGTACWACACCTTC	
			GROEL-F#	TGGCAAATGTAGTTGTAACAGG	
			GROEL-R#	GCCGACTTTTAGTACAGCAA	
	semi-nested PCR	*fts*Z	Eh_ftsZ_703_R1*	CWGCTTCTCCTGTRCCCATCAT	[68]
			Eh_ftsZ_313_F2#	ACTGCYGGAATGGGTGGWGGA	
			Eh_ftsZ_679_R2*#	TTTRCCCATYTCRCTCATTATTGC	
	nested PCR	conP28	conP28-F1	AT(C/T)AGT(G/C)AAA(A/G)TA(T/C)(A/G)T(G/A)CCAA	[68]
			conP28-R1	TTA(G/A)AA(A/G)G(C/T)AAA(C/T)CT(T/G)CCTCC	
			conP28-F2	CAATGG(A/G)(T/A)GG(T/C)CC(A/C)AGA(AG)TAG	
			conP28-R2	TTCC(T/C)TG(A/G)TA(A/G)G(A/C)AA(T/G)TTTAGG	

	PCR	*dsb*	Eh-dsb-330 F	GATGATGTCTGAAGATATGAAACAAAT	[69]
			Eh-dsb-728 R	CTGCTCGTCTATTTTACTTCTTAAAGT	
Borrelia spp.	qPCR	23S rRNA	Bo23S-F	GAGTCTTAAAAGGGCGATTTAGT	[70]
			Bo23S-R	CTTCAGCCTGGCCATAAATAG	
			Bo23S-Probe	AGATGTGGTAGACCCGAAGCCGAGT	
	nested PCR	*osp*A	Bor-ospA-F1*	GGGAATAGGTCTAATATTAGCC	[53]
			Bor-ospA-R1*	CACTAATTGTTAAAGTGGAAGT	
			Bor-ospA-F2#	GCAAAATGTTAGCAGCCTTGAT	
			Bor-ospA-R2#	CTGTGTATTCAAGTCTGGC	
Francisella tularensis	qPCR	*fop*A	Fr-fopA-F	GGCAAATCTAGCAGGTCAAGC	[70]
			Fr-fopA-R	CAACACTTGCTTGAACATTTCTAG	
			Fr-fopA-Probe	AACAGGTGCTTGGGATGTGGGTGGTG	
Coxiella burnetii	qPCR	IS*1111*	Co-IS111-F	TGGAGGAGCGAACCATTGGT	[70]
			Co-IS111-R	CATACGGTTTGACGTGCTGC	
			Co-IS111-Probe	ATCGGACGTTTATGGGGATGGGTATCC	
Bartonella	qPCR	*ssr*A	Bar-ssrA-F	GCTATGGTAATAAATGGACAATGAAATAA	[71]
			Bar-ssrA-R	GCTTCTGTTGCCAGGTG	
			Bar-ssrA-Probe	ACCCCGCTTAAACCTGCGACG	
Babesia–Theileria–Hepatozoon	nested PCR	18S rRNA	BTH18S-F1*	GTGAAACTGCGAATGGCTCATTAC	[33]
			BTH18S-R1*	AAGTGATAAGGTTCACAAAACTTCCC	
			BTH18S- F2#	GGCTCATTACAACAGTTATAGTTTATTTG	
			BTH18S- R2#	CGGTCCGAATAATTCACCGGAT	

*Primers using in first round of nested PCR or semi-nested PCR

Primers using in second round of nested PCR or semi-nested PCR
